# Parenting stress during infancy is a risk factor for mental health problems in 3-year-old children

**DOI:** 10.1186/s12889-020-09861-5

**Published:** 2020-11-16

**Authors:** Nayantara Hattangadi, Katherine T. Cost, Catherine S. Birken, Cornelia M. Borkhoff, Jonathon L. Maguire, Peter Szatmari, Alice Charach

**Affiliations:** 1 Department of Psychiatry, Hospital for Sick Children, Toronto, ON Canada; 2 Division of Pediatric Medicine and the Paediatric Outcomes Research Team (PORT), Hospital for Sick Children, Toronto, ON Canada; 3 Child Health Evaluative Sciences, SickKids Research Institute, Toronto, ON Canada; 4 Department of Pediatrics, Faculty of Medicine, University of Toronto, Toronto, ON Canada; 5 Institute of Health Policy Management and Evaluation, University of Toronto, Toronto, ON Canada; 6 Joannah & Brian Lawson Centre for Child Nutrition, Department of Nutritional Sciences, University of Toronto, Toronto, ON Canada; 7 Women’s College Research Institute, Women’s College Hospital, Toronto, ON Canada; 8 The Centre for Urban Health Solutions, Li Ka Shing Knowledge Institute of St. Michael’s Hospital, Toronto, ON Canada; 9 Department of Pediatrics, St. Michael’s Hospital, Toronto, ON Canada; 10 Department of Psychiatry, University of Toronto, Toronto, ON Canada; 11 Centre for Addiction and Mental Health, Toronto, ON Canada

**Keywords:** Parenting stress, Child mental health, Prospective cohort, Mental health, Parent-child relationship

## Abstract

**Background:**

Although research on the relationship between parent and child mental health is growing, the impact of early parenting stress on preschool-aged children’s mental health remains unclear. The objective was to evaluate the association between parenting stress during infancy and mental health problems in 3-year-old children.

**Methods:**

A prospective cohort study of healthy preschool-aged children recruited from 9 primary care practices in Toronto, Canada was conducted through the TARGet Kids! primary care practice-based research network. Parenting stress was measured when children were between 0 to 16 months of age, using the Parent Stress Index Short Form, PSI-SF. Parent-reported child mental health problems were measured at 36 to 47 months using the preschool Strengths and Difficulties Questionnaire, total difficulties score (TDS). Hierarchical linear regression analysis was used to investigate the association between standardized PSI-SF and TDS, adjusted for child age, sex, temperament, sleep duration and household income. To strengthen clinical interpretation, analysis was repeated using adjusted multivariable logistic regression (TDS dichotomized at top 20%).

**Results:**

A total of 148 children (mean ± SD age, 37.2 ± 1.7 months, 49% male) were included in the analysis. Parenting stress during infancy (11.4 ± 3.1 months of age) was significantly associated with mental health problems in 3-year-old children (β = 0.35; 95% CI = 0.20–0.49, *p* < 0.001). Higher parenting stress was also associated with increased odds of higher TDS (OR = 2.26, 95% CI = 1.69–2.83, *p* < 0.01).

**Conclusion:**

Healthy preschool-aged children with parents reporting parenting stress during infancy had a 2 times higher odds of mental health problems at 3 years.

## Background

The etiology of mental health problems in young children has garnered growing research and clinical interest (1). Early childhood is a period marked by critical emotional, social and behavioral development; in the early years, mental health problems can manifest as internalizing behaviors such as fearfulness and social withdrawal, and externalizing behaviors such as irritability, temper outbursts and oppositional, non-compliant behavior (2, 3). Young children with such concerns continue to have difficulties as they grow older; over time, these difficulties are associated with poor academic, employment and health trajectories (4, 5).

Our understanding of early modifiable risk factors for mental health problems in preschool-aged children is incomplete. In older children, parenting stress has been reported as a risk factor for parent and child psychopathology (6, 7). Although the literature is limited, some studies have explored the impact of parenting stress in children under 10 years of age and have found links with child behavior problems (8, 9). However, previous studies focused on parenting stress in the context of children with neurodevelopmental difficulties, chronic illnesses, or developmental delays (10, 11). Thus, the impact of early parenting stress on typically developing preschool-aged children is unclear.

There are confounding factors that impact the relationship between parenting stress and mental health problems in preschool-aged children that should be considered. Namely, temperament or biologically-based differences in child behavior patterns, have been identified as early risk factors of mental health problems in children (12, 13). In particular, high negative affect, evidenced as irritability, and low effortful control or poor impulse self-control, have been linked to subsequent diagnoses of Attention Deficit Hyperactivity Disorder (ADHD), Oppositional Defiant Disorder (ODD), anxiety disorders and depression (14, 15). As well, children who have high scores on inhibition are at a greater risk of anxiety disorders than those with lower scores on this temperamental domain (14). Moreover, there is increasing interest in the relationship between sleep and mental health in this age group (16, 17). Some studies link child sleep problems, bedtime resistance, and parental perceptions of child’s sleep as problematic, with higher parenting stress (18).

The relationship between parenting stress in infancy and development of mental health problems in preschool-aged children is understudied. Parenting stress is a potentially modifiable factor that may contribute to mental health problems in young children. Findings could lead to including regular assessments of parenting stress as part of developmental assessments for children, thereby informing practitioners to assist parents with managing and alleviating parenting stress, and to identify and support children at higher risk for mental health problems.

The objective of our study was to evaluate the association between early parenting stress and mental health problems in preschool-aged children.

## Methods

### Participants

We conducted a prospective study of healthy urban children recruited during health supervision visits from primary care practices participating in The Applied Research Group for Kids! (TARGetKids!), a community-based research network in Toronto, Canada (www.targetkids.ca) (19). Trained research assistants administer parents/caregivers standardized questionnaires to collect information on child and parental health (19). In 2016, the preschool Strengths and Difficulties Questionnaire (P-SDQ), a brief parent-report screening measure identifying internalizing and externalizing behaviors in young children was added to the TARGet Kids! study protocol (20).

**Fig. 1:**
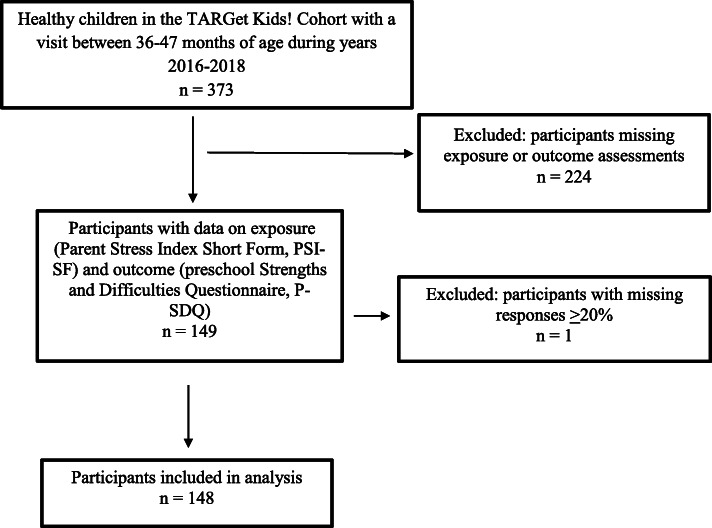
Participant flowchart 1

Children were included in this study if their parent/caregiver completed the measure of parenting stress (Parent Stress Index Short Form; PSI-SF) during infancy (baseline, between 0 to 16 months of age), as well as the P-SDQ during the 3 year health supervision visit (outcome, between 36 to 47 months of age). In addition, parent report of 24-h sleep duration and temperament (Child Behavior Questionnaire, Very Short Form; CBQ-VSF), collected during the 3 year health supervision visit, were also required for inclusion in this study. TARGet Kids! exclusion criteria are: < 32 weeks gestational age; health conditions affecting growth (e.g. cystic fibrosis); chronic conditions (other than asthma and high functioning autism); severe developmental delay; unscheduled visit because of acute illness; and families who are unable to complete the measures in English (19). A total of 149 children met the inclusion criteria for this study (Fig. 1). One participant had > 20% of missing responses and was excluded, leaving *n* = 148 for analysis.

Informed written consent was obtained by parents, and ethical approval was granted from the Research Ethics Boards at The Hospital for Sick Children and St. Michael’s Hospital, Toronto, Canada. The cohort study is registered at www.clinicaltrials.gov (identifier NCT01869530).

### Exposure variable: parenting stress

PSI-SF is a valid and reliable parent-report measure for early identification of parent–child systems under excessive stress (21). It has 36-items, representing three domains: parent distress, parent-child dysfunctional interaction, and difficult child. Parenting stress scores, based upon all three domains, range from 36 to 180. Parenting stress percentile scores that fall between 15 and 80 are considered typical; over 80th percentile is high. The parenting stress score has excellent internal consistency in our sample (Cronbach’s alpha: 0.95).

### Outcome variables: mental health problems

The P-SDQ is a 25-item parent-report measure used to identify young children at high risk for mental health disorders. The P-SDQ has good psychometric properties and is valid for use with children age 2–4 years (22). It is divided into five subscales (emotional problems, conduct problems, hyperactivity/inattention, peer problems and prosocial behavior), each comprising of five items rated on a 3-point Likert scale (0–2), with scores ranging from 0 to 10. The total difficulties score (TDS) includes all subscale scores except the prosocial behavior subscale, and ranges from 0 to 40. In our sample, the total difficulties score has acceptable internal consistency (Cronbach’s alpha: 0.70). The total difficulties score can be dichotomized as being at risk of clinical problems (TDS > 12) or not at risk (< 12). Cutoff was determined based on published norms and measures for borderline and definite clinical risk, as well as identifying scores for the top 20% (characterized as being at risk of clinical problems) in our total sample of 3-year-old children (23, 24).

### Covariates

An a priori literature search was conducted to identify potential confounds in the relationship between early parenting stress and mental health problems in preschool-aged children. Covariates identified included age at baseline, sex, temperament, sleep duration, and household income. Parent-reported temperament was measured using the CBQ-VSF (25). CBQ-VSF is a valid and reliable scale that identifies three domains: negative affect (negative emotional reactions), effortful control (self-control) and surgency/extraversion (motor activity and seeking closeness with others). We included the negative affect and effortful control temperament domains as confounders, as there is substantial literature in support of their impact on emotional and behavioral problems in young children (13–15). In our sample, negative affect and effortful control have acceptable internal consistency (Cronbach’s alpha: 0.75 and 0.69, respectively). Twenty-four hour sleep duration was reported by parents in response to the question “How many hours does your child usually spend sleeping in a 24-hour period”? Sleep duration was parameterized as a continuous variable denoting the number of hours slept in a 24-h period. Child age and sex, and self-report annual household income, were collected during enrollment.

### Sample size

The sample size calculation for this study was based on a review of the existing literature on parenting stress and mental health problems in young children. A study with a small effect size (*r* = 0.1) and a power of 80% would require a minimum sample of 128, to detect an association at an alpha level of 5% (26). Thus, our sample of 148 was deemed appropriate.

### Statistical analysis

Descriptive statistics were calculated for the exposure, outcomes, and covariates and presented as mean values (standard deviations) for continuous data, and percentages for categorical data. A correlation matrix was conducted to assess the Pearson correlations between key variables in this study. Hierarchical linear regression analysis was conducted to examine the association between early parenting stress and mental health problems, using a three step-approach: 1) with covariates (model 1); 2) including key temperament variables (model 2); and 3), adding parenting stress (model 3). In addition, we used adjusted multivariable logistic regression analysis to strengthen clinical interpretation of the relationship between our exposure and outcome variable (total difficulties score dichotomized at top 20%). All variables were standardized to interpret logistic regressions as 1 standard deviation change. Covariates specified a priori (age at baseline, sex, negative affect, effortful control, sleep duration, and household income) were included in the adjusted multivariable models. Statistical analysis was performed using R version 3.6.1 (27). Significance level was set at *p* < 0.05.

Twelve of 148 participants had some responses missing on their PSI-SF (< 9%). Missing responses were imputed using multiple imputation. We created and analyzed 10 imputed datasets (28, 29). Missing values on household income (1%) were also imputed.

## Results

Table 1 outlines descriptive characteristics. The mean (±SD) age of children was 11.4 (±3.1) months at baseline, and 37.2 (±1.7) months at outcome; 49% of children included in this study were male. Parents scored an average of 60.0 ± 15.5 on parenting stress, well below scores considered high. Approximately 2% of parenting stress percentile scores fell within the high or clinically significant range. Parents were relatively well educated and financially secure as 96% of mothers had a college/university degree, and 94% reported an annual household income >$60,000.

**Table 1:** Descriptive Characteristics

Characteristics	All participants (***n*** = 148)
**Age, mo, mean ± SD (min-max)**	
Baseline	11.4 **±** 3.1 (0–16.0)
Outcome	37.2 **±** 1.7 (36.0–47.0)
**Male, n (%)**	73 **(**49.3)
**Mental Health Problems**^**a**^**, mean ± SD (min-max)**	8.8 **±** 4.4 (1.0–26.0)
**Parenting stress**^**b**^**, mean ± SD (min-max)**	60.0 **±** 15.5 (36.0–105.0)
**Temperament**^**c**^**, mean ± SD (min-max)**	
Negative affect	3.7 **±** 0.9 (1.8–6.1)
Effortful control	5.4 **±** 0.7 (3.5–6.7)
**Sleep duration**^**d**^**, mean ± SD (min-max)**	11.6 **±** 1.1 (8.0–14.0)
**Maternal education high school or less, n (%)**	5 (3.4)
**Household income**^**e**^ **< $60,000, n (%)**	7 (4.7)

*SD* standard deviation

^a^Total difficulties score - preschool Strengths and Difficulties Questionnaire (P-SDQ)

^b^Total stress score – Parent Stress Index Short Form (PSI-SF)

^c^Child Behavior Questionnaire – Very Short Form (CBQ-VSF)

^d^Number of hours slept in a 24-h period

^e^Annual household income (dichotomized at 60,000 CAD)

A statistically significant correlation was identified between parenting stress and mental health problems (Pearson correlation coefficient = 0.44, 95% CI = 0.29–0.56, *p* = 0.001). Significant correlations between parenting stress and negative affect, as well as between both temperament traits and mental health problems, were also found. See Table 2.

**Table 2:** Correlation Matrix

Variable	1	2	3	4
1. Mental health problems	1.0	0.41*	−0.37*	0.44*
2. Negative affect	0.41*	1.0	−0.10	0.38*
3. Effortful control	−0.37*	−0.10	1.0	−0.01
4. Parenting stress	0.44*	0.38*	−0.01	1.0

*p < 0.05

After accounting for child age at baseline, sex, household income, and sleep duration (model 1), negative affect and effortful control (model 2), parenting stress during infancy (model 3) was associated with total mental health problems in children at 3 years (β = 0.35; 95% CI = 0.20–0.49, *p* < 0.001). Model 3 explained significant variance in the outcome (adjusted R-squared = 0.39, F (7,140) = 14.35, *p* < 0.001) and significantly more variance than Model 1 (adjusted R-squared = 0.06, F (4,143) = 3.67, *p* = < 0.01) and Model 2 (adjusted R-squared = 0.29, F (6,141) = 11.46, *p* < 0.001). See Table 3.

**Table 3:** Hierarchical linear regression for association between parenting stress and mental health problems in 3-year-old children

Variable	Model 1		Model 2		Model 3	
	*β* (log)	95% CI	*β* (log)	95% CI	*β* (log)	95% CI
Age^a^	−0.01	− 0.06, 0.04	− 0.01	− 0.06, 0.03	− 0.03	−0.07, 0.01
Sex, female	−0.28	−0.60, 0.03	− 0.16	−0.45, 0.12	− 0.25	−0.52, 0.02
Household income,^b^ low	1.19*	0.45, 1.94	0.89*	0.24, 1.55	0.50	−0.13, 1.14
Sleep duration	0.01	−0.15, 0.17	−0.01	− 0.15, 0.13	−0.00	− 0.13, 0.13
Negative affect			0.34*	0.21, 0.49	0.23*	0.09, 0.37
Effortful control			−0.31*	−0.46, − 0.17	−0.31*	− 0.45, − 0.18
Parenting Stress					0.35*	0.20, 0.49
Adjusted R^b^	0.06*		0.29*		0.39*	
Model F-statistic (df)	3.67 (4, 143)		11.46 (6, 141)		14.35 (7, 140)	
Change in F-statistic (*p*-value)	0.007		< 0.001		< 0.001	

*CI* indicates Confidence interval

^a^Age at baseline

^b^Annual household income dichotomized at 60,000 CAD (> 60,000 = high)

*p < 0.05.

In addition, a one standard deviation increase in parenting stress during infancy was associated with twice the odds of the child being at high risk of total mental health difficulties at 3 years (OR = 2.26, 95% CI = 1.69–2.83, *p* < 0.01). See Table 4**.**

**Table 4:** Adjusted logistic regression for association between parenting stress and mental health problems in 3-year-old children^a^

Variable	Adjusted OR	95% CI
Age^b^	0.91	0.77, 1.04
Sex, female	0.96	0.02, 1.94
Household income^c^, low	3.17	1.23, 5.10
Sleep duration	0.99	0.45, 1.52
Negative affect	1.67*	1.15, 2.18
Effortful Control	0.44*	0.08, 0.94
Parenting stress	2.26*	1.69, 2.83

^a^ adjusted for all other variables in Table

^b^Age at baseline

^c^Annual household income dichotomized at 60,000 CAD (> 60,000 = high)

*OR* indicates Odds ratio, *CI* indicates Confidence interval

*p < 0.05.

## Discussion

We found that stress in the parent-child system during infancy was associated with subsequent mental health problems in preschool-aged children. Indeed, while parenting stress during infancy is associated with negative affect, its contribution to preschool mental health problems is beyond that of temperament factors known to be linked. This finding expands our understanding of modifiable predictors during infancy and toddlerhood, and suggests that parenting stress is an important potential target for intervention (30).

Conceptually, the observed relationship between parent-child systems under stress and emotional and behavioral problems in children is well described. Parents under stress are less likely to be engaged, more irritable and distant with their young children (31). Particularly in the early years, positive bonding experiences and interactions between parent and child are fundamental for building competencies that support growth and development. A lack of early parental nurturance, parental negativity or over-reactivity can impact emotional and behavioral development in children, and have deleterious long-term consequences including, but not limited to, negative adjustment outcomes (e.g. social-emotional and peer difficulties), and increased risk for mental health and substance use problems as they grow older (32, 33).

In addition, it is important to highlight the bidirectional associations between parenting stress and child behavior. Stress can negatively affect parents’ interpretations of child behaviors, shaping subsequent parent-child interactions, potentially reducing parental responsiveness and sensitivity towards children, thus, impacting child behavior (34, 35). While the associations are difficult to disentangle, it is also well established that excessive stress that hinders parents’ abilities to appropriately manage their own moods and reactions can perpetuate ongoing difficulties for parents and their children (36). Finally, this relationship could be reflective of genetic variants that affect both a vulnerability to experience stress in parents, and emotional and behavioral problems in their children (37).

To our knowledge, much of the existing research has been completed in clinical settings (e.g. parents have post-partum psychosis or children are experiencing psychiatric disorders); this study is novel as it is documenting the association between parenting stress during infancy and mental health problems in a typically developing population. The impact of early parenting stress reinforces that mental health interventions for children must regularly assess parental well-being and include programs to assist parents in managing parenting-related stress in order to effectively benefit young children. In addition, regular assessment of parenting stress can support clinicians in identifying preschool children at higher risk of clinical mental health problems. Mental health interventions with parenting programs (aimed at improving parent-child interactions, managing parenting stress, and promoting mindful parenting) can be valuable contributions to early mental health management in families (38, 39). Some of these early interventions have identified improvements in child internalizing and externalizing behaviors, as well as reductions in parenting stress (40).

### Strengths and limitations

A strength of this study was the prospective analysis of parenting stress during infancy, and subsequent assessment of mental health problems in typically developing 3-year-old children. An additional strength of this study was controlling for several important covariates, such as temperament traits, sleep duration and sociodemographic characteristics. Due to our primary care setting, our study was limited by reliance on parent-report measures, which could have common method bias. Parenting stress can affect parents’ perceptions of child behavior, and it is possible that parents who experience early parenting stress are more likely to perceive their preschool child’s behavior as problematic. Future research could include measures completed by additional caregivers, as well as more intensive observational data collection such as clinical assessments. In addition, few families that participated in our study experience high levels of socioeconomic risk and are largely of middle-higher socio-economic status. Therefore, the results may not be generalizable to less-advantaged populations. Similarly, most of the reports of parenting stress measured in our study are considered typical. Yet, the relative lack of financial stressors and clinically significant parenting stress strengthens the importance of our findings – even in relatively affluent families, and among parents experiencing typical parenting stress, early parenting stress is associated with child mental health outcomes in preschool years.

## Conclusion

Mental health problems in children emerge early and can have adverse and long-lasting impact on social, emotional and behavioral outcomes. This study highlights the role of early parenting stress as a risk factor for mental health problems in preschool-aged children. Our initial findings support further exploration of the relationship among parenting stress, child temperament and the emergence of mental health problems in early childhood. Study findings also underline the importance of identifying parents experiencing stress to help ameliorate mental health problems in preschool-aged children.

## Data Availability

The datasets used and/or analyzed during the current study are available upon reasonable request by contacting www.targetkids.ca/contact-us/.

## Abbreviations

P-SDQ: Preschool Strengths and Difficulties Questionnaire
PSI-SF: Parent Stress Index Short Form
CBQ-VSF: Child Behavior Questionnaire – Very Short Form
